# Anti-cyclic citrullinated peptide antibodies and paraoxonase-1 polymorphism in rheumatoid arthritis

**DOI:** 10.1186/1471-2474-15-379

**Published:** 2014-11-19

**Authors:** Hassan El-Banna, Asif Jiman-Fatani

**Affiliations:** Department of Medical Microbiology and Parasitology, Faculty of Medicine, King Abdulaziz University, Jeddah, Saudi Arabia

**Keywords:** Rheumatoid arthritis, Anti-CCP-2 antibodies, Paraoxonase-1 polymorphism, Rheumatoid factor, Arylesterase

## Abstract

**Background:**

Rheumatoid arthritis (RA) is the most common chronic inflammatory joint disease, with a worldwide prevalence of 0.5% to 1%. Anti-cyclic citrullinated peptide antibody (anti-CCP-2 Ab) is a marker of choice for diagnosing early and late RA. Anti-oxidant enzymes activity decreases in RA patients. Till now, the relationship between the rheumatoid factor (RF) and anti-CCP-2 Ab, anti-oxidant activity and polymorphism of paraoxenase-1 (PON-1) 192 Q/R in patients with RA has not been investigated. In this study, we aimed to determine the serum level of RF and anti-CCP-2 Ab, PON-1 activity and 192 Q/R polymorphism and arylesterase (ARE) activity in patients with RA. Also, we studied RA markers in different genotypes of PON-1 of RA patients.

**Methods:**

A total of 120 RA patients and 90 healthy persons were subjected to full clinical examinations and routine laboratory tests. PON-1 and ARE activities were determined using an enzymatic spectrophotometric method. PON-1 192 gene polymorphism was determined using polymerase chain reaction based restriction fragment analysis. RF was measured by immunoturbidimetry method and anti-CCP-2 Ab was assayed by enzyme-linked immunosorbent assay (ELISA). Statistical analysis was performed using SPSS for windows 20.0.

**Results:**

The sensitivity and specificity of anti-CCP-2 Ab for the diagnosis of RA were 76.2% and 100% respectively. PON-1 and ARE activities were statistically lower (P <0.001) in the RA group compared to the control group. A negative correlation between RF and anti-CCP-2 Ab levels and PON-1 and ARE activities was found. No significant difference in the genotype distribution between RA patients and healthy persons was detected. RF and anti-CCP-2 Ab levels were higher in RA patients carried RR genotype than in those carried QQ genotype.

**Conclusion:**

High RF and anti-CCP-2 antibody serum levels were found to be associated with decreased PON-1 and ARE activities with no correlation between PON-1 polymorphism and serum levels of RF and anti-CCP-2 Ab in patients with RA. These results may indicate an implication between antioxidant enzymes activity and serum levels of RF and anti-CCP-2 Ab.

## Background

Rheumatoid arthritis (RA) is a chronic multisystem disease of unknown cause characterized by chronic polyarthritis and can lead to deformities and disabilities. Early intervention is crucial in preventing irreversible joint damage, so it is important to diagnose RA at very early stage in the disease [1]. When often not all clinical symptoms are manifested, so, good serological markers are needed. Anti-CCP-2 Ab is a marker of choice for diagnosing early RA as it appears to be highly specific and sensitive for the disease comparable to other tests such as RF test. Furthermore, anti-CCP-2 Ab positivity can predict future development of RA in both asymptomatic individuals and in patients with undifferentiated arthritis. Moreover, its antibody level at presentation can correlate with progression to erosive disease [2].

Although the exact pathogenesis of the disease is unknown, both genetic and environmental factors play key roles in the disease process [3]. It has been reported that reactive oxygen species (ROS) may play an important role in the pathogenesis of RA [4, 5]. Under normal circumstances, relatively low concentrations of ROS are formed in all cells and tissues in oxidative processes and a variety of antioxidative mechanisms serve to control their production [6]. Under pathological conditions, the levels of ROS are altered by increased production and/or inadequate removal by antioxidants such as paraoxonase enzyme (PON).

The paraoxonases (PON) gene family consists of three members; PON-1, PON-2, and PON-3 located adjacent to each other on the long arm of human chromosome number 7. These three human PON genes share approximately 60% identity at the amino acid level and approximately 70% identity at the nucleotide level [7]. Human serum PON-1 is a calcium dependent glycoprotein which is tightly associated with high density lipoprotein HDL [8]. PON-1 decreases accumulation of the lipid peroxides in low density lipoprotein (LDL) due to its ability to reduce ROS and attenuates the biological effects of mildly oxidized LDL [9]. PON-1 serum activity appears to be different among individuals and populations. The molecular basis of such variations may be due to polymorphism in PON-1 gene. PON-1 has two polymorphisms at the coding region; one in position 192, which is a glutamine/arginine substitution and the other at position 55 which is a methionine/leucine substitution [10]. These polymorphisms may affect the hydrolytic activity of PON- 1 isoenzymes with respect to lipid peroxidation. It has been reported that paraoxonase activity of the glutamine 192 (Q allele) isoforms is lower than that of the arginine 192 (R allele) isoforms [11]. Nevertheless, PON-1 in QQ subjects seems to be more effective in protecting LDL from oxidation than that in RR genotype subjects. Although the relationship between PON-1 192 activity and polymorphism in rheumatoid arthritis was investigated, the correlation between the serum levels of RF and anti-CCP-2 Ab and PON-1 192 activity and polymorphism is not cleared. So, the main aim of the present work was designed to investigate the relationship between gene PON-1 192 polymorphism and serum PON-1 and ARE activities and the serum levels of RF and anti-CCP-2Ab in RA patients.

## Methods

### Patients

This study was carried out on 210 individuals, classified into two groups. Group I: 120 patients with RA with duration of the disease ranged from 4 to 12 years. They were 90 females (75%) and 30 males (25%). Their ages ranged from 18 to 62 years with a mean value of 41.33 ± 15.93 years. The patients fulfilled the 1987 revised American College of Rheumatology criteria for RA. Patients were selected from the out patients’ clinics at different Jeddah’s hospitals including King Abdulaziz University Hospital. Group II: 90 healthy controls without joint complaints or any rheumatological disease; 60 females (66.66%) and 30 males (33.33%). Their ages ranged from 19 to 64 years with a mean value of 40.5 ± 17.56 years.

### Exclusion criteria

Patients having diseases overlapping with other connective tissue diseases like systemic lupus erythematosus (SLE), systemic sclerosis, polymyositis and those suffering from renal diseases, hypertension, ischemic heart disease, HIV and smokers were excluded from the study. Patients were subjected to complete history taking, full clinical examinations and radiological and routine laboratory investigations. Patients underwent; clinical assessment of disease activity by full history taking, general and local examinations, measurement of the 28-joint count of tender and swollen joints with calculation of the disease activity score (DAS-28) for each RA patient by DAS-28 score calculator. Written informed consents were obtained from all patients and controls according to the Declaration of Helsinki and the study was approved ethically by Committee of the Department of Medical Microbiology and Parasitology, Faculty of Medicine, King Abdulaziz University, Jeddah, Kingdom of Saudi Arabia.

### Sampling

Blood samples were collected from all patients and controls and divided into 2 parts; one for genetic study and the other part of the plasma was separated and stored at −70°C for serological and enzymatic studies. The sample for genetic study was put in sterile K_3_EDTA (tri-potassium ethylenediamine tetraacetic acid) coated tubes which were used for the DNA extraction. White cells were removed from the buffy coat for DNA extraction. Samples were stored at −20°C till the time of use.

### Laboratory tests

#### a- Determination of rheumatoid factor (RF)

RF was measured by immunoturbidimetry using Cobas integra RFII (Roche Diagnostics GmbH, Mannheim, Germany) according to the instructions of the manufacturer. Positivity is ≥20 IU/ml.

#### b- Determination of serum anti-CCP- 2 antibodies

Anti-CCP-2 Ab was detected by ELISA (DIASTAT Axis-Shield, Dundee, United Kingdom) according to the instructions of the manufacturer. Positivity is ≥5 U/ml.

#### c- Determination of serum PON-1 activity

PON-1 activity was measured by adding plasma to Tris buffer (100 mmol/l, pH 8.0) containing 2 mmol/l CaCl_2_ and 1 mmol/l paraoxon (O, O-diethyl-O-nitrophenylphosphate (Sigma). The rate of generation of P-nitrophenol was determined at 405 nm, 25°C with the use of continuously recording spectrophotometer as described previously [12, 13].

#### d- Determination of serum arylesterase (ARE) activity

Arylesterase (ARE) activity was measured using phenylacetate as a substrate as previously described [14]. The initial rates of hydrolysis were determined by following the increase of phenol concentration at 270 nm at 37°C on a CE 7250 spectrophotometer (Cecil Instruments Limited, UK). Enzyme activities were expressed in international units per 1 liter of serum (U/l).

#### e- Determination of PON-1 genotypes

DNA was extracted from peripheral leukocytes using sodium dodecyl sulphate (SDS) lysis and ethanol precipitation [15]. Aliquots of genomic DNA were used for PCR amplification as described previously [16, 17]. Two primers, the sense primer: 5′ TAT TGT TGC TGT GGG ACC TGA G 3′ and antisense primer: 5′ CAC GCT AAA CCC AAA TAC ATC TC 3′ were used for PCR amplification of 99 bp DNA fragment covering the region containing Gln192 or Arg 192. The PCR reaction mixture contained about 200 ng DNA template, 0.5 μM of each primer, 1.5 mM MgCl_2_, 200 μM of the 4 dNTP and 1 U Taq DNA polymerase (Amersham, Bioscience). After denaturing the DNA for 4 minutes at 95°C, the reaction mixture was subjected to 35 cycles of denaturing for 1 minute at 95°C, 1 minute annealing at 60°C and 1 minute extension at 72°C. The 99 bp PCR products were digested with 8 U Alw1 restriction endonuclease (MBI Fermentas) overnight at 37°C and the digested products were separated by electrophoresis on 2% agarose electrophoresis and visualized using ethidium bromide. The Arg genotype contains a unique Alw1 restriction site which results in 66 and 33 bp products, whereas the Gln genotype will not be cleaved by this restriction enzyme.

### Statistical analysis

This was performed using SPSS for windows 20.0 (SPSS Inc, Chicago. IL, USA). Student’s *t* test was used to compare mean values of continuous variables in cases and controls, whereas *χ*^2^ analysis was used to compare categorical data. Correlation between variables was evaluated using Pearson (r) correlation coefficient. Relative risk was performed to examine genotype risk contribution.

## Results

Table 1 showed that the ages of patients were matched with ages of controls. The ratio of female to male in RA patients was more than that in controls. As regarding ESR, RF PON-1, ARE activities and anti-CCP-2 Ab there was significant changes in RA patients when compared to controls.

**Table 1:** **Demographic pattern and laboratory results of RA patients and controls**

Parameters	RA patients	Controls	***t***-test	P value
**Number (F/M)**	120 (90/30)	90 (60/30)	-	NS
**Age (years)**	41.33 ± 15.93	40.5 ± 17.5	0.36	NS
**Duration of illness (years)**	9.65 ± 4.28	0.0	-	-
**ESR (1st hr) mm/hr**	41.62 ± 8.02	8.65 ± 2.89	17.21	0.001*
**RF** **Mean ± SE (IU/mL)**	(149.34 ± 64.30)	13.5 ± 21	18.99	0.001*
**Anti-CCP-2 Ab** **Mean ± SE IU/mL**	62.54 ± 21.22	4.41 ± 1.20	19.85	0.001*
**PON-1 activity (nmol/min/ml)**	64.44 ± 11.33	152.18 ± 17.74	16.25	0.001*
**ARE activity (Um/l)**	197.65 ± 83.84	363.65 ± 169.15	10.52	0.001*

Data are presented as mean ± SD. NS means not significant.

P value was calculated by *t*-test P* value was significant at <0.001.

The clinical characteristics of the 120 RA patients are shown in Table 2. Patients had a moderate-active disease as shown by the high DAS (4.18 ± 1.73) and HAQ (1.6 ± 0.64) scores. Moreover, one hundred and five patients were treated by (disease-modifying anti-rheumatic drugs (DMARDs). Overall, 95 patients were receiving steroid therapy.

**Table 2:** **Characteristics of RA patients**

Parameters (mean ± SD)	RA patients
**Positive RF, n (%)**	65 (54.28%)
**Positive Anti-CCP-2 Ab, n (%)**	83 (69.57%)
**Current DMARDs therapy, n (%)**	105 (87.5%)
**Current steroid therapy, n (%)**	95 (79.4%)
**Tender joints count (n)**	16.6 ± 8.8
**Swollen joints count (n)**	9.8 ± 5.6
**DAS28**	4.18 ± 1.73
**Pain VAS score**	51.25 ± 20.09
**HAQ score**	1.6 ± 0.64

Values are reported as mean **±** SD.

DMARDs: disease-modified anti-rheumatic drugs.

DAS28: disease activity score in 28 joints.

VAS: visual analog scale.

HAQ: Health Assessment Questionnaire.

Table 3 showed a negative correlation between PON-1 and ARE enzymatic activity and serum levels of RF and anti-CCP-2 Abs.

**Table 3:** **Correlation coefficient study between enzymes activity and RA biomarkers**

Parameter	Test	ARE activity (U/l)	RF (IU/ml)	Anti-CCP-2 (U/ml)
**PON-1 activity (nmol/min/ml)**	*r*	*0.66*	−0.77	−0.85
	*P*	0.001*	0.001*	0.001*
**ARE (U/l)**	*r*		0.23	0.36
	*p*		0.156	0.18
**RF (IU/ml)**	*r*			0.77
	*P*			>0.001*

P* value was significant at <0.001.

Table 4 showed no significant difference in distribution of QQ, QR and RR genotypes between both RA patients and controls. However, there was a significant correlation between PON-1 activity and genotypes. Also, Table 4 showed Q and R allele’s frequency in PON-1 in the RA patients which was 76.26% for Q allele and 23.74% for R allele; meanwhile, it was 74.44% for Q and 25.56% for R in controls. RR and OR equations did not show a significant risk between RA patients and controls.

**Table 4:** **Genotype and allele frequency among RA patients and controls**

Genotype	Patients (n = 120)	Controls (n = 90)	P value	Relative risk	O.R.
**QQ**	70% (84/120)	68.90% (62/90)	0.862*	1.02	1.05
**RR**	17.5% (21/120)	20% (18/90)	0.644*	0.93	0.85
**QR**	12.5 (15/120)	11.10% (10/90)	0.758*	1.06	1.14
	**Allele frequency**				
**Q**	183 (76.26%)	134 (74.44%)	0.670*	1.04	1.10
**R**	57 (23.74%)	46 (25.56%)			

P value was calculated by *χ* ^2^ -test.

P* value was not significant (P>0.05).

Table 5 showed a significant increase of PON-1 activity, serum levels of RF and anti-CCP-2 Ab in RA patients carried RR genotype compared to QQ genotype. While regarding the ARE activity; no significant difference was detected in RA patients with RR alleles compared to QQ genotype.

**Table 5:** **PON-1, ARE activities, RF and Anti-CCP-2 Ab in QQ, QR and RR genotypes in the RA patients**

Test	Q Q	Q R	R R	P value
	No = 84	No = 21	No = 15	
**PON-1 activity (nmol/min/ml)**	56.75 ± 8.62	71.63 ± 6.11	78.56 ± 11.83	0.001*
**ARE activity (U/l)**	181.21 ± 80.56	223.34 ± 81.94	178.52 ± 72.09	0.098
**RF (IU/ml)**	149.54 ± 35.96	159.40 ± 32.54	188.75 ± 26.81	0.003*
**Anti-CCP-2 Ab (U/ml)**	46.91 ± 20.95	62.32 ± 14.73	79.42 ± 12.11	0.0021*

Data are presented as mean ± SD. NS means not significant.

P value was calculated by students -test.

P* value was significant at P <0.05.

## Discussion

In the present work, the diagnostic value of antibodies anti-CCP-2 Ab and RF in the diagnosis of rheumatoid arthritis (RA) was evaluated. Furthermore, the relationship between the gene polymorphism of PON-1 and its enzyme activity, ARE enzyme activity and serum levels of RF and anti-CCP-2 Ab were investigated. In our study, the mean concentration of RF was 149.34 ± 64.30 IU/ml and 54.76% of the RA patients were sero-positive. The serum concentrations of RF were more than that reported by Mansour et aI [18] and similar to that of Kim et al. [19]. The sensitivity and specificity of RF were 30% and 70% respectively. Our results did not match with the results of Choi at all and Greiner et al. [20, 21]. This can be attributed to the different methods used for RF measurement, selection of cases, disease durations and administration of different drugs.

Anti-CCP-2 antibody is a marker of choice for diagnosing early RA as it appears to be highly specific for the disease. Furthermore, the emerging data strongly suggest that anti-CCP-2 antibodies have the power to predict the development of RA in patients with early and undifferentiated arthritis, the severity of disease in patients with established RA and the possibility of future onset of RA in certain high-risk populations [22]. In our study, the mean concentration of anti-CCP-2 Abs was 62.54 ± 21.22 U/mL in all RA patients (71.8% were sero-positive) which was similar to that detected by Kim et al. [19] and more than what was detected by Al-Shukaili et al. [23]. As regards anti-CCP-2 Ab sensitivity and specificity, they were 76.2% and 100% respectively. Also, several authors [24, 25] reported similar percentages. However, Berrocal et al. and Nielen et al. [26, 27] found lower sensitivities and specificities for anti-CCP-2 (43% and 85%) and (57.8% and 94.2%) respectively. Such differences can be explained by using different ELISA kits with different cut off values. Anti-CCP-2 Ab serum levels have an additional diagnostic value over RF in RA patients [28, 29]. Another evidence for superiority of anti-CCP-2 Ab as a good diagnostic marker in Saudi patients with RA in comparison with RF came from the study of Safi et al. [30] who found that a proportion (30.5%) of RF-negative RA patients, had positive anti-CCP-2 Ab serum levels. However, the IgM-RF is still widely used as a screening marker in the diagnosis of RA. Moreover, anti-CCP-2 Ab assays have a comparable sensitivity in the diagnosis of RA but with a much higher specificity. The anti-CCP-2 Ab assay has proved to be very helpful and seems to be the diagnostic marker of choice for the diagnosis of RA especially for early cases [2, 29]. The increase in the serum level of anti-CCP-2 Abs is correlated with the duration of RA and complications [31].

In the present study, PON-1 and ARE activities were decreased significantly in RA patients compared with the controls. Such results are in basic agreement to the previous reports of Altinadg et al., Baskol et al., Isik et al. and Tanimoto et al., [32–35]. The low PON-1 and ARE enzymes activities in RA patients may result from the damage of PON-1 and ARE proteins by the action of high amount of reactive oxygen species produced in RA patients rather than reduced synthesis [34]. Furthermore, we demonstrated the relationship between RF, anti-CCP-2 Abs, PON-1 and ARE where we found a negative correlation between anti-CCP-2 Ab and antioxidative enzymes activities of PON-1 and ARA activity. Therefore, our study is unique in the investigation of the relationship between RF and anti-CCP-2 Ab tests and PON-1 and ARE enzymes. It has been suggested that anti-CCP-2 Ab may be related to the increase oxidative activity in RA patients [36, 37]. For example, TNF-alpha inhibitor acts as a regulator against pentosidine formation, oxidative DNA damage and lipid peroxidation and is associated with a decrease in serum levels of oxidative stress markers, and also anti-CCP-2 Abs levels in RA patients [38, 39]. In a similar way; reducction of PON-1 activity may lead to elevation of anti-CCP-2 Ab. Reduction of PON-1 activity may aggravate ROS formation leading to more lipid peroxidation, cellular and DNA damage consequently resulting in more formation of anti-CCP-2 Ab.

In this study, there was no significant difference in the distribution of Q and R alleles between the RA patients and controls. By using risk factor equation and Odds ratio we could not able to detect any risk factor between the RA patients and controls. Therefore, PON-1 Q192R polymorphism might not be associated with the incidence of rheumatoid arthritis among Saudi population. This result was in basic agreement with the work of Hashemi et al. [40] who found no significant differences between RA Iranian patients and controls. Tanimoto et al. [35] found that there was a difference in the distribution of PON-1 192 Q/R genotypes among RA Japanese patients and healthy subjects. PON-1 activity was obviously low among RA patients who carried PON-1 Q genotype compared with that in healthy subjects despite the absence of differences in PON-1-R between the two groups. This discrepancy might be due to the difference in ethnic groups. In the present study, the QQ genotype was the major prevailing type in our patient group with a 70% percentage followed by QR (17.5%) then RR (12.5%). The Q allele frequency was significantly higher than R allele in both RA patients and controls. The Q allele carriers were previously reported to be protected against peroxidation more than R allele carriers [11].

The present study showed a significant difference in PON-1 activity among RR genotype compared to QQ and QR genotypes i.e. RR genotype was the most active towards paraoxon, QQ genotype was the least active, while the activity of QR genotype was intermediate. However, no difference was detected in ARE enzyme activity among QQ, QR and RR genotypes in RA patients. Tripi et al. [41] indicated that the PON-1 Q192R polymorphism has substantial influence on PON-1 activity towards paraoxon, which has been reported by many other investigators.

Our results showed a significant elevation of the serum levels of RF and anti-CCP-2 Ab in RR genotype compared to QQ genotype. This novel unique observation was not demonstrated in previous known studies. The occurrence of high serum levels of RF and anti-CCP-2 Abs which had been detected more in RR genotype than in QQ genotype can be explained by the following five points: (1) It has been suggested that there is an association between the high serum levels of anti-CCP-2 Ab and increased oxidative stress in RA patients [39]. (2) The increase of anti-CCP-2 Ab serum level is strongly associated with both prevalent tissue erosions and the development of erosions in RA joint tissues. (3) High serum levels of anti-CCP-2 Abs have been associated with an erosive disease outcome in RA. (4) RA synovial fluid and tissue have also demonstrated oxidative damage to hyaluronic acid, low-density-lipoproteins (LDL), proteins, cartilage, extracellular collagen, and intra-cellular DNA. (5) The PON-l R allele appears to have a lower capacity to protect against oxidation of tissue rather than the PON-1Q allele consequently; one may suggest that the R allele, has a low capacity to protect tissue from oxidation than that of PON- 1 Q allele.

## Conclusions

In conclusion, there is a reduction in PON-1 and ARE activities in RA patients. Also, we found a relationship between high serum levels of RF and anti-CCP-2 Ab and decreased PON-1 and ARE enzymatic activities in RA patients. Furthermore, it has been found that there was no association between Q192R polymorphism of PON-1 and RA. Moreover, it has been found a significant increase of RF and anti-CCP-2 Ab levels in RR genotype compared to QQ genotype.

## Authors’ information

Prof Dr. Hassan El-banna**,** MBChB, MSc, PhD (Virology): Professor of Medical Microbiology, Department of Medical Microbiology and Parasitology, Faculty of Medicine, King Abdulaziz University, Jeddah, Kingdom of Saudi Arabia. Prof El-Banna has a wide experience in the field of Clinical Microbiology. He is a member of departmental committee. Also, he is a member of many specialized societies. Prof El-Banna has many publications in the field of Microbiology and Virology. The main interest of Prof El-Banna is in the field of Clinical Microbiology and Immunology.

Dr. Asif A. Jiman-Fatani, MBChB, MSc, PhD (UK) (Medical Microbiology) is an Associate Professor of Medical Microbiology. He is the Chairman of the Department of Medical Microbiology and Parasitology, Faculty of Medicine, King Abdulaziz University. Dr. Jiman-Fatani is a Consultant Microbiologist. He is the Head of the Clinical & Molecular Microbiology Laboratory, King Abdulaziz University Hospital (KAUH). Also, he is a member of the Infection Control Committee at KAUH. He is a member of the Saudi Society of Medical Microbiology and Infectious Diseases. He has many publications in the field of Medical Microbiology and Immunology. The main interest of Dr. Jiman-Fatani is in the field of Clinical Microbiology and Immunology.
